# γ-Aminobutyric acid (GABA) and ectoine (ECT) impacts with and without AMF on antioxidants, gas exchange attributes and nutrients of cotton cultivated in salt affected soil

**DOI:** 10.1186/s12870-023-04486-3

**Published:** 2023-10-09

**Authors:** Yuhan Ma, Ping Huang, Shoucheng Huang, Uzma Younis, Ghulam Sabir Hussain, Shah Fahad, Subhan Danish, Mohamed Soliman Elshikh, Humaira Rizwana

**Affiliations:** 1 College of Life and Health Science, Anhui Science and Technology University, Fengyang, 233100 China; 2 College of Chemistry and Materials Engineering, Anhui Science and Technology University, Fengyang, 233100 China; 3 Botany Department, The Islamia University of Bahawalpur, Sub Campus Rahim Yar Khan, Rahim Yar Khan, Punjab, Pakistan; 4 Department of Agronomy, Faculty of Agricultural Sciences and Technology, Bahauddin Zakariya University, Multan, 66000 Pakistan; 5 Department of Agronomy, Abdul Wali Khan University Mardan, Mardan, Khyber Pakhtunkhwa, Mardan, 23200 Pakistan; 6 Department of Soil Science, Faculty of Agricultural Sciences and Technology, Bahauddin Zakariya University, Multan, Pakistan; 7 Department of Botany and Microbiology, College of Science, King Saud University, P.O. 2455, 11451 Riyadh, Saudi Arabia

**Keywords:** Osmoprotectants, Salinity stress, Cotton, Growth attributes, Chlorophyll contents

## Abstract

Salinity stress is one of the major hurdles in agriculture which adversely affects crop production. It can cause osmotic imbalance, ion toxicity that disrupts essential nutrient balance, impaired nutrient uptake, stunted growth, increased oxidative stress, altered metabolism, and diminished crop yield and quality. However, foliar application of osmoprotectant is becoming popular to resolve this issue in crops. These osmoprotectants regulate the cellular osmotic balance and protect plants from the detrimental effects of high salt concentrations. Furthermore, the role of arbuscular mycorrhizae (AMF) is also established in this regard. These AMF effectively reduce the salinity negative effects by improving the essential nutrient balance via the promotion of root growth. That’s why keeping in mind the effectiveness of osmoprotectants current study was conducted on cotton. Total of six levels of γ-Aminobutyric acid (GABA = 0 mM, 0. 5 mM, and 1 mM) and ectoine (ECT = 0 mM, 0.25 mM, and 0.5 mM) were applied as treatments in 3 replications. Results showed that 0.5 mM γ-Aminobutyric acid and ectoine performed significantly best for the improvement in cotton growth attributes. It also caused significant enhancement in K and Ca contents of the leaf, stem, bur, and seeds compared to the control. Furthermore, 0.5 mM γ-Aminobutyric acid and ectoine also caused a significant decline in Cl and Na contents of leaf, stem, bur, and seeds of cotton compared to control under salinity stress. A significant enhancement in chlorophyll contents, gas exchange attributes, and decline in electrolyte leakage validated the effectiveness of 0.5 mM γ-Aminobutyric acid and ectoine over control. In conclusion, 0.5 mM γ-Aminobutyric acid and ectoine have the potential to mitigate the salinity stress in cotton.

## Introduction

Salinity stress is a significant abiotic factor [1] that adversely affects crop growth and productivity [2–5]. When soil or water contains high concentrations of salts, particularly sodium chloride, it can impede plant development and result in numerous negative consequences [6–9]. The detrimental effects of salinity stress on crops encompass reduced water uptake due to osmotic imbalance, ion toxicity that disrupts essential nutrient balance [4, 10], impaired nutrient uptake, stunted growth, increased oxidative stress, altered metabolism, and diminished crop yield and quality [11]. Excess salts hinder water absorption by plants, leading to dehydration and water stress [12, 13]. Furthermore, the disturbed nutrient balance and reduced availability of essential nutrients [14, 15].

To overcome this issue, the use of osmoprotectants as foliar application is becoming popular. γ-Aminobutyric acid (GABA) and ectoine (ECT) are two such examples of osmoprotectants that have shown potential in mitigating the effects of salinity stress on crop plants [16]. GABA is a non-protein amino acid that accumulates in plant cells under various stress conditions, including salinity stress [17]. It plays a crucial role as an Osmo protectant by regulating cellular osmotic balance and protecting plants from the detrimental effects of high salt concentrations [18]. GABA acts as a signaling molecule and interacts with different metabolic pathways to enhance stress tolerance in plants [19]. It can scavenge reactive oxygen species, stabilize membranes, and modulate ion transport, thereby maintaining cellular integrity and function [20].

Ectoine is a naturally occurring osmoprotectant produced by halophilic microorganisms [21]. It can stabilize cellular structures and maintain proper cell function under high salinity conditions [22]. Ectoine acts as a compatible solute by protecting proteins, enzymes, and membranes from salt-induced denaturation and dehydration [23]. Its application as a foliar spray has demonstrated potential in enhancing salt stress tolerance in plants by reducing oxidative damage, maintaining photosynthetic activity, and promoting overall plant growth and productivity [24]. Furthermore, arbuscular mycorrhizal fungi (AMF) have emerged as valuable allies for plants facing salinity stress in their growth environment [25]. The mutualistic relationship between AMF and plant roots provides several benefits, especially under conditions of high salt concentrations [26]. AMF enhances plant tolerance to salinity stress through various mechanisms [16]. They improve nutrient uptake by extending their hyphal networks into the soil, accessing otherwise inaccessible nutrients [27].

Cotton is one of the most economically important fiber crops globally, providing raw material for the textile industry [28, 29]. It is cultivated in various regions, including areas with high soil salinity, where salinity stress poses a significant challenge to cotton production [30]. Salinity stress adversely affects cotton plant growth, reduces yield, and impairs fiber quality [31]. While the effects of salinity stress on cotton crops and the potential use of osmoprotectants have been studied to some extent, there is a knowledge gap in understanding the specific mechanisms and efficacy of osmoprotectant application for mitigating salinity stress in cotton plants [32].

The current study is covering the regarding the use of γ-Aminobutyric acid (GABA) and ectoine (ECT) combination against salinity stress. The study aimed to examine the impact of γ-Aminobutyric acid (GABA) and ectoine (ECT) on cotton cultivated under salinity stress. We hypothesized that the foliar application of osmoprotectants might enhance the salinity stress tolerance of cotton crops. The osmoprotectants, through their osmotic adjustment properties and antioxidant activities, are expected to improve water uptake, maintain ion homeostasis, and mitigate oxidative damage in cotton plants exposed to salinity stress.

## Material and methods

### Plant material

Cotton seeds (CIM 616 BT) were used for the experiment. Cotton seeds were surface sterilized with a suitable sterilizing agent (e.g., 5% sodium hypochlorite) and rinsed thoroughly with sterile distilled water [33]. The sterilized seeds were germinated in sterilized sand or paper towels in a controlled environment with appropriate temperature and humidity until the seedlings reached a uniform size. Uniform 2 seedlings were transferred for further experimentation in the pot.

### Pot preparation

Plastic pots of a specific size (10 inch × 18 inch) were filled with a saline soil substrate. In each pot, 15 kg of soil was filled. Before filling the pot debris and clods of soil were removed manually by using a sieve. The soil used was representative of the target growing conditions and was salt-affected. The characteristic of the soil is provided in Table 1.

**Table 1:** Pre-experimental soil and irrigation characteristics

Soil	Values	References	Irrigation	Values	References
pH	8.34	[34]	pH	7.15	[35]
EC*e* (dS/m)	5.64	[36]	EC (µS/cm)	712	
SOM (%)	0.50	[37]	Carbonates (meq./L)	0.00	
TN (%)	0.03	[38]	Bicarbonates (meq./L)	4.19	
AP (µg/g)	2.10	[39]	Chloride (meq./L)	0.015	
EK (µg/g)	61	[40]	Ca + Mg (meq./L)	3.0	
ENa (µg/g)	456	[41]	Sodium (mg/L)	131	
Texture	Loam	[42]	TN = Total Nitrogen; AP = Available Phosphorus; EK = Extractable Potassium ENa = Extractable Sodium		

### Arbuscular mycorrhizal fungi (AMF)

To introduce arbuscular mycorrhizal fungi (AMF) into the soil, a commercial inoculum (Clonex® Root Maximizer, 5711 Enterprise Drive, Lansing, MI, USA) was utilized. The inoculum consisted mainly of Glomus species and had a concentration of 158 propagules per gram. For the experimental setup, 3.75 g of the inoculum were added to 15 kg of soil [43]. For inoculation, AMF inoculum was evenly distributed across the soil's surface in a sterilized container. A thorough mixing followed, ensuring the AMF inoculum was uniformly incorporated into the soil. The moisture of the soil was kept at 65% field capacity. Finally, the soil mixture was transferred to pots as per the treatment plan.

### GABA and ECT

The γ-Aminobutyric acid (GABA) and ectoine (ECT) were prepared at different concentrations by suing analytical grade salts i.e., GABA = Product Number: A2129; Batch Number: BCCJ0874; Color: White; Appearance = Powder; Sigma Aldrich and ECT = Product Number: 81619; Batch Number: BCCJ4305; Color: White; Appearance = Powder; Sigma Aldrich. For the GABA treatments (0 mM, 0.5 mM, and 1 mM), the solutions were foliar applied to the plants at a rate of 25 mL per pot, five times at 15, 17, 19, 21, and 24 days after transplantation. For the ECT treatments (0 mM, 0.25 mM, and 0.5 mM), the solutions were also foliar applied at the same rate and frequency.

### Experimental design

The experiment followed a randomized complete block design (RCBD) with a factorial arrangement of treatments. All treatments were applied in 3 replicates in salt-affected soil. The treatment plant is provide in Table 2.

**Table 2:** Treatment plan

Treatment	AMF	GABA and ECT
T1	No AMF	0 mM GABA
T2	No AMF	0.5 mM GABA
T3	No AMF	1 mM GABA
T4	No AMF	0 mM ECT
T5	No AMF	0.25 mM ECT
T6	No AMF	0.5 Mm ECT
T7	AMF	0 mM GABA
T8	AMF	0.5 mM GABA
T9	AMF	1 mM GABA
T10	AMF	0 mM ECT
T11	AMF	0.25 mM ECT
T12	AMF	0.5 Mm ECT

*mM* miliMolar

### Fertilizer

Nitrogen was applied in three equal splits, with a total rate of 115 kg ha^−1^, at 40, 60, and 80 days after planting. The nitrogen source used was urea. Phosphorus, at a rate of 60 kg ha^−1^, was applied in its diammonium phosphate (DAP) form, while potassium, also at a rate of 60 kg ha^−1^, was supplied as potassium sulfate (K_2_SO_4_). The complete dose of phosphorus and potassium was broadcasted at the time of sowing to ensure availability to the growing cotton crop [44].

### Irrigation

A total of nine irrigations were administered during the cotton sowing season, with the timing of each irrigation based on the soil moisture levels. The initial irrigation was provided at the time of planting, and subsequent irrigations were scheduled based on the soil moisture content monitoring [44].

### Data collection

Soon after harvesting, the following parameters were analyzed to assess the effects of the treatments: germination (%), plant height (cm), stem dry biomass (g/pot), leaf dry biomass (g/pot), boll dry biomass (g/pot), seed dry biomass (g/pot), shed dry biomass (g/pot), root dry biomass (g/pot).

### Digestion of samples for nutrient analysis

One gram sample was subjected to a diacid digestion mixture (10 mL). The diacid digestion protocol was employed, which involved the use of a diacid mixture consisting of concentrated nitric acid (HNO_3_) and concentrated perchloric acid (HClO_4_) in 2:1 ratio. The digestion process was initiated by heating the vessels in a digestion block or furnace at a specific temperature, typically around 180 °C -200 °C until the solution become clear like water [45].

### Nutrients analysis

The flame photometer was calibrated using standard solutions of known concentrations for each element (K, Ca, and Na) [41]. Calibration curves were generated by measuring the emission intensities of the standards at specific wavelengths. For Cl analysis, a few drops of potassium chromate (K_2_CrO_4_) indicator solution were added to the flask. After that titration of the sample solution was done with standard silver nitrate (AgNO_3_) solution until a reddish-brown color appeared. To convert milliequivalents per liter (meq/L) to grams per kilogram (g/kg) [35].

$$1\mathrm{ meq}/\mathrm{L }= (\mathrm{molar mass in g}/\mathrm{mole }/\mathrm{ valence}) \times 1\mathrm{ mg}/\mathrm{L }\times 1\mathrm{ g}/\mathrm{kg}$$

$$1\mathrm{ meq}/\mathrm{L }= (35.45\mathrm{ g}/\mathrm{mole }/\mathrm{ valence}) \times 1\mathrm{ mg}/\mathrm{L }\times 1\mathrm{ g}/\mathrm{kg}$$

$$1\mathrm{ meq}/\mathrm{L }= 35.45\mathrm{ mg}/\mathrm{L }= 35.45\mathrm{ g}/\mathrm{kg}$$

### Chlorophyll contents and gas exchange attributes

The chlorophyll a, chlorophyll b, and total chlorophyll contents in fresh wheat leaves were determined following the protocol described by Arnon [46]. Leaf extracts were obtained using an 80% acetone solution. To estimate the chlorophyll a and chlorophyll b contents, the absorbance of the extracts was measured at specific wavelengths using a spectrophotometer. The absorbance at 663 nm was recorded for chlorophyll a, while the absorbance at 645 nm was recorded for chlorophyll b. Using the obtained absorbance values, the final calculations for chlorophyll a, chlorophyll b, and total chlorophyll content were performed using the following relationships:

$$\mathrm{Chlorophyll a }\left(\frac{\mathrm{mg}}{\mathrm{g}}\right)= 12.7\left(\mathrm{OD }663\right)-2.69\left(\mathrm{OD }645\right)\times \mathrm{V}/1000 (\mathrm{W})$$

$$\mathrm{Chlorophyll b }\left(\frac{\mathrm{mg}}{\mathrm{g}}\right)= 22.9\left(\mathrm{OD }645\right)-4.68\left(\mathrm{OD }663\right)\times \mathrm{V}/1000 (\mathrm{W})$$

$$\mathrm{Total Chlorophyll }\left(\frac{\mathrm{mg}}{\mathrm{g}}\right)= 20.2\left(\mathrm{OD }645\right)+8.02\left(\mathrm{OD }663\right)\times \mathrm{V}/1000 (\mathrm{W})$$

The photosynthetic rate, transpiration rate, and stomatal conductance of plant samples were analyzed using an Infrared Gas Analyzer (IRGA) (CI-340 Photosynthesis system, CID, Inc. USA). On a sunny day, the readings were taken between 10:30 AM and 11:30 AM at the saturating intensity of light [47, 48].

### Electrolyte leakage

The electrolyte leakage (EL) was assessed using a modified version of the method described by Lutts et al. [49]. To determine EL, the leaves were carefully washed with deionized water to remove any external contaminants. Subsequently, uniform-sized leaf pieces weighing approximately one gram were excised using a steel cylinder with a diameter of 1 cm. These leaf pieces were placed in individual test tubes containing 20 ml of deionized water and incubated at a temperature of 25 °C for a period of 24 h. After the incubation period, the electrical conductivity (EC1) of the water solution in the test tubes was measured using an EC meter that had been pre-calibrated. The test tubes were then subjected to a water bath set at 120 °C for 20 min, and the electrical conductivity (EC2) was recorded following the heating process.

$$\mathrm{EL }(\mathrm{\%}) = (\mathrm{EC}1 /\mathrm{ EC}2) \times 100$$

### Antioxidants

Superoxide dismutase (SOD) activity was determined by measuring the inhibition of nitro blue tetrazolium (NBT) reduction in the presence of riboflavin. The reaction mixture, consisting of enzyme extract, NBT, riboflavin, and phosphate buffer, was illuminated, and the change in absorbance at 560 nm [50]. Peroxidase (POD) activity was evaluated by monitoring the oxidation of a suitable substrate, such as guaiacol or o-dianisidine [51]. Catalase (CAT) activity was determined by monitoring the decomposition of hydrogen peroxide (H_2_O_2_) by the enzyme [52]. The decrease in absorbance at 240 nm resulting from H_2_O_2_ decomposition was measured. Ascorbate peroxidase (APX) activity was assessed by monitoring the oxidation of ascorbate in the presence of H_2_O_2_ [53].

### Statistical analysis

The collected data was subjected to standard statistical analysis [54]. Means were compared using paired comparison by applying Fisher’s LSD test at a significance level of *P* < 0.05. For making graphs, cluster plot convex hull and hierarchical cluster plot OriginPro 2021 software was used [55].

## Results

### Germination

**Fig. 1:**
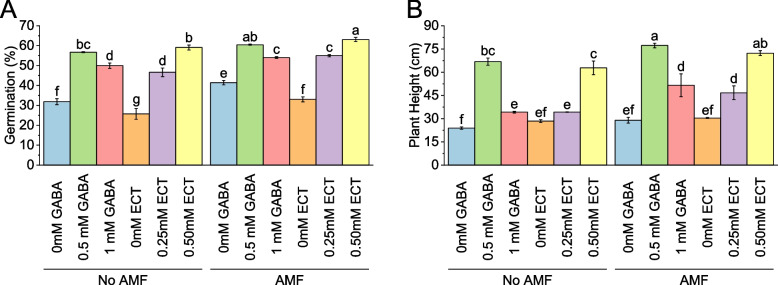
Effect of osmoprotectants γ-Aminobutyric acid (GABA) and ectoine (ECT) different foliar application rates with and with AMF on germination (**A**) and plant height (**B**) of cotton cultivated in salinity stress (soil EC = 5.64 dS/m). Bars are means of 3 replicates ± SE. Different letters on bars showed significant changes at p ≤ 0.05; Fisher’s LSD

When no arbuscular mycorrhizal fungi (AMF) were present and 0 mM gamma-aminobutyric acid (GABA) was used as an osmoprotectant, the germination percentage was 31.84%. With 0.5 mM GABA, the germination percentage increased to 56.73%, representing a 78.19% increase compared to the control. When 1 mM GABA was used, the germination percentage was 49.93%, showing a 56.81% increase. In the absence of AMF and using 0 mM ectoine (ECT) as an osmoprotectant, the germination percentage was 25.71%. However, when 0.25 mM ECT was introduced, the germination percentage significantly increased to 46.61%, indicating an 81.29% increase. Using 0.5 0 mM ECT resulted in a germination percentage of 59.08%, representing a remarkable 129.83% increase compared to the control. On the other hand, when AMF were present and 0 mM GABA was used as the osmoprotectant, the germination percentage was 41.43%. With 0.5 mM GABA, the germination percentage increased to 60.48%, reflecting a 45.96% increase. When 1 mM GABA was used, the germination percentage was 54.02%, showing a 30.39% increase compared to the control. In the presence of AMF and using 0 mM ECT as an osmoprotectant, the germination percentage was 33.04%. However, when 0.25 mM ECT was introduced, the germination percentage significantly increased to 54.97%, indicating a 66.39% increase. Using 0.50 mM ECT resulted in a germination percentage of 63.09%, representing a notable 90.95% increase compared to the control (Fig. 1A).

### Plant height

In the case of no arbuscular mycorrhizal fungi (AMF) and 0 mM gamma-aminobutyric acid (GABA) was used as an osmoprotectant, the average plant height was 23.91 cm. With 0.5 mM GABA, the plant height significantly increased to 66.79 cm, indicating a 179.36% increase compared to the control. When 1 mM GABA was used, the average plant height was 34.19 cm, showing a 43.02% increase. Without AMF and using 0 mM ectoine (ECT) as an osmoprotectant, the average plant height was 28.45 cm. However, when 0.25 mM ECT was introduced, the plant height increased to 34.23 cm, indicating a 20.34% increase. Using 0.50 mM ECT resulted in an average plant height of 62.81 cm, representing a significant 120.79% increase compared to the control. In AMF and 0 mM GABA was used as the osmoprotectant, the average plant height was 28.96 cm With 0.5 mM GABA, the plant height increased to 77.25 cm, reflecting a 166.76% increase. When 1 mM GABA was used, the average plant height was 51.56 cm, showing a 78.04% increase compared to the control. For AMF and 0 mM ECT, the average plant height was 30.42 cm. However, when 0.25 mM ECT was introduced, the plant height increased to 46.72 cm, indicating a 53.57% increase. Using 0.50 mM ECT resulted in an average plant height of 72.34 cm, representing a notable 137.80% increase compared to the control (Fig. 1B).

### Leaf dry biomass

**Fig. 2:**
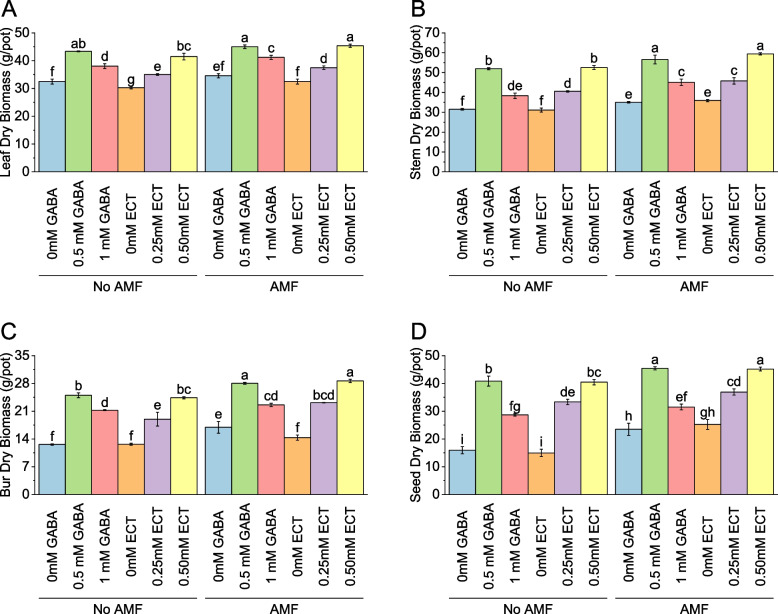
Effect of osmoprotectants γ-Aminobutyric acid (GABA) and ectoine (ECT) different foliar application rates with and with AMF on leaf dry biomass (**A**), stem dry biomass (**B**), bur dry biomass (**C**) and seed dry biomass (**D**) of cotton cultivated in salinity stress (soil EC = 5.64 dS/m). Bars are means of 3 replicates ± SE. Different letters on bars showed significant changes at p ≤ 0.05; Fisher’s LSD

In the absence of AMF, the control group (0 mM GABA) exhibited a mean leaf dry biomass of 32.48 g/pot. Interestingly, the introduction of 0.5 mM GABA led to a significant increase in leaf dry biomass, with a mean value of 43.38 g/pot, representing a 33.58% enhancement compared to the control. Similarly, the use of 1 mM GABA resulted in a mean leaf dry biomass of 38.06 g/pot, signifying a 17.20% increase. When AMF were absent and ECT was employed as the osmoprotectant, the mean leaf dry biomass for the control group (0 mM ECT) was 30.26 g/pot. However, the introduction of 0.25 mM ECT led to a notable increase in leaf dry biomass, with a mean value of 35.04 g/pot, indicating a 15.79% enhancement. Furthermore, the use of 0.50 mM ECT resulted in a mean leaf dry biomass of 41.47 g/pot, reflecting a substantial increase of 37.05% compared to the control. In contrast, when AMF were present, the control group (0 mM GABA) exhibited a mean leaf dry biomass of 34.58 g/pot. The introduction of 0.5 mM GABA significantly increased the leaf dry biomass to 45.03 g/pot, representing a 30.22% enhancement. Similarly, the use of 1 mM GABA resulted in a mean leaf dry biomass of 41.19 g/pot, indicating a 19.12% increase compared to the control. Under the presence of AMF and using 0 mM ECT as the osmoprotectant, the mean leaf dry biomass was 32.49 g/pot. However, the introduction of 0.25 mM ECT led to a significant increase in leaf dry biomass, with a mean value of 37.48 g/pot, signifying a 15.35% enhancement. Moreover, the use of 0.50 mM ECT resulted in a mean leaf dry biomass of 45.38 g/pot, representing a substantial increase of 39.68% compared to the control (Fig. 2A).

### Stem dry biomass

For AMF, the control group (0 mM GABA) exhibited a mean stem dry biomass of 31.52 g/pot. Notably, the introduction of 0.5 mM GABA resulted in a significant increase in stem dry biomass, with a mean value of 51.97 g/pot, representing a 64.92% enhancement compared to the control. Similarly, the use of 1 mM GABA yielded a mean stem dry biomass of 38.34 g/pot, signifying a 21.64% increase. Without AMF and with ECT as the osmoprotectant, the control group (0 mM ECT) displayed a mean stem dry biomass of 31.13 g/pot. However, the addition of 0.25 mM ECT led to a notable increase in stem dry biomass, with a mean value of 40.57 g/pot, indicating a 30.30% enhancement. Moreover, the utilization of 0.50 mM ECT resulted in a mean stem dry biomass of 52.59 g/pot, reflecting a substantial increase of 68.93% compared to the control. Conversely, with AMF, the control group (0 mM GABA) showed a mean stem dry biomass of 35.06 g/pot. The introduction of 0.5 mM GABA significantly increased the stem dry biomass to 56.60 g/pot, representing a 61.44% enhancement. Similarly, the utilization of 1 mM GABA yielded a mean stem dry biomass of 45.14 g/pot, indicating a 28.74% increase compared to the control. In the presence of AMF and employing 0 mM ECT as the osmoprotectant, the mean stem dry biomass was 35.93 g/pot. Nevertheless, the inclusion of 0.25 mM ECT led to a significant increase in stem dry biomass, with a mean value of 45.81 g/pot, signifying a 27.49% enhancement. Furthermore, the use of 0.50 mM ECT resulted in a mean stem dry biomass of 59.42 g/pot, representing a notable increase of 65.34% compared to the control (Fig. 2B).

### Bur dry biomass

Under no AMF, the control group (0 mM GABA) exhibited a mean bur dry biomass of 12.62 g/pot. Remarkably, the introduction of 0.5 mM GABA resulted in a substantial increase in bur dry biomass, with a mean value of 24.99 g/pot, representing a remarkable 98.07% increase compared to the control. Similarly, the utilization of 1 mM GABA led to a mean bur dry biomass of 21.25 g/pot, signifying a significant 68.37% increase. In the absence of AMF and using ECT as the osmoprotectant, the control group (0 mM ECT) displayed a mean bur dry biomass of 12.66 g/pot. However, the introduction of 0.25 mM ECT resulted in a notable increase in bur dry biomass, with a mean value of 18.98 g/pot, indicating a 50.00% enhancement. Furthermore, the utilization of 0.50 mM ECT yielded a mean bur dry biomass of 24.39 g/pot, reflecting a substantial increase of 92.69% compared to the control. Under AMF, the control group (0 mM GABA) showed a mean bur dry biomass of 16.93 g/pot. The introduction of 0.5 mM GABA significantly increased the bur dry biomass to 28.04 g/pot, representing a 65.57% enhancement. Similarly, the utilization of 1 mM GABA yielded a mean bur dry biomass of 22.59 g/pot, indicating a 33.38% increase compared to the control. In the case of AMF with 0 mM ECT as the osmoprotectant, the mean bur dry biomass was 14.35 g/pot. However, the inclusion of 0.25 mM ECT led to a significant increase in bur dry biomass, with a mean value of 23.15 g/pot, signifying a 61.35% enhancement. Furthermore, the use of 0.50 mM ECT resulted in a mean bur dry biomass of 28.63 g/pot, representing a remarkable increase of 99.57% compared to the control (Fig. 2C).

### Seed dry biomass

For no AMF with 0 mM GABA, the mean seed dry biomass was 15.92 g/pot. However, the introduction of 0.5 mM GABA resulted in a substantial increase in seed dry biomass, with a mean value of 40.85 g/pot, representing a notable percentage increase of 156.58 compared to the control. Similarly, the utilization of 1 mM GABA yielded a mean seed dry biomass of 28.67 g/pot, indicating a significant percentage increase of 80.06. In the case of no AMF with ectoine (ECT) as the osmoprotectant, the control group (0 mM ECT) displayed a mean seed dry biomass of 14.97 g/pot. However, the introduction of 0.25 mM ECT led to a substantial increase in seed dry biomass, with a mean value of 33.34 g/pot, signifying a remarkable percentage increase of 122.73. Furthermore, the utilization of 0.50 mM ECT resulted in a mean seed dry biomass of 40.46 g/pot, reflecting a significant percentage increase of 170.31 compared to the control. For AMF, the control group (0 mM GABA) showed a mean seed dry biomass of 23.48 g/pot. The introduction of 0.5 mM GABA significantly increased the seed dry biomass to 45.44 g/pot, representing a notable percentage increase of 93.53. Similarly, the utilization of 1 mM GABA yielded a mean seed dry biomass of 31.52 g/pot, indicating a percentage increase of 34.24 compared to the control. In AMF with 0 mM ECT as the osmoprotectant, the mean seed dry biomass was 25.22 g/pot. However, the inclusion of 0.25 mM ECT led to a significant increase in seed dry biomass, with a mean value of 36.91 g/pot, signifying a percentage increase of 46.35. Furthermore, the use of 0.50 mM ECT resulted in a mean seed dry biomass of 45.18 g/pot, representing a substantial percentage increase of 79.12 compared to the control (Fig. 2D).

### Shed dry biomass

**Fig. 3:**
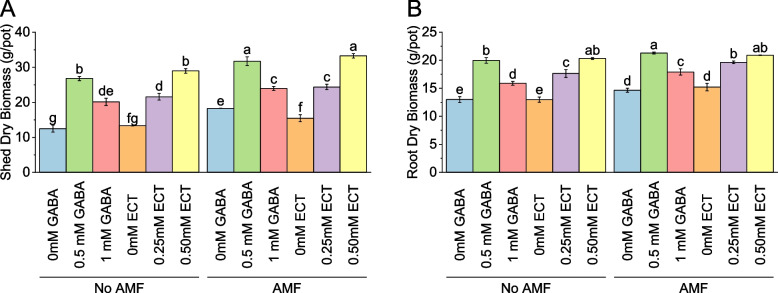
Effect of osmoprotectants γ-Aminobutyric acid (GABA) and ectoine (ECT) different foliar application rates with and with AMF on shed dry biomass (**A**) and root dry biomass (**B**) of cotton cultivated in salinity stress (soil EC = 5.64 dS/m). Bars are means of 3 replicates ± SE. Different letters on bars showed significant changes at p ≤ 0.05; Fisher’s LSD

In the absence of arbuscular mycorrhizal fungi (AMF) and with 0 mM gamma-aminobutyric acid (GABA) used as the osmoprotectant, the mean shed dry biomass was 12.51 g/pot. However, the introduction of 0.5 mM GABA resulted in a significant increase in shed dry biomass, with a mean value of 26.81 g/pot, representing a substantial percentage increase of 114.32 compared to the control. Similarly, the utilization of 1 mM GABA yielded a mean shed dry biomass of 20.12 g/pot, indicating a notable percentage increase of 60.87. Under the absence of AMF and with ectoine (ECT) as the osmoprotectant, the control group (0 mM ECT) displayed a mean shed dry biomass of 13.32 g/pot. However, the introduction of 0.25 mM ECT led to a significant increase in shed dry biomass, with a mean value of 21.59 g/pot, signifying a notable percentage increase of 62.04. Furthermore, the utilization of 0.50 mM ECT resulted in a mean shed dry biomass of 28.98 g/pot, reflecting a substantial percentage increase of 117.56 compared to the control. Conversely, in the presence of AMF, the control group (0 mM GABA) showed a mean shed dry biomass of 18.28 g/pot. The introduction of 0.5 mM GABA significantly increased the shed dry biomass to 31.72 g/pot, representing a notable percentage increase of 73.58. Similarly, the utilization of 1 mM GABA yielded a mean shed dry biomass of 23.97 g/pot, indicating a percentage increase of 31.15 compared to the control. Under the presence of AMF and employing 0 mM ECT as the osmoprotectant, the mean shed dry biomass was 15.46 g/pot. However, the inclusion of 0.25 mM ECT led to a significant increase in shed dry biomass, with a mean value of 24.38 g/pot, signifying a notable percentage increase of 57.70. Furthermore, the use of 0.50 mM ECT resulted in a mean shed dry biomass of 33.28 g/pot, representing a substantial percentage increase of 115.23 compared to the control (Fig. 3A).

### Root dry biomass

Without arbuscular mycorrhizal fungi (AMF), the mean root dry biomass was 13.00 g/pot when no gamma-aminobutyric acid (GABA) was added as an osmoprotectant. However, the introduction of 0.5 mM GABA led to a significant increase in root dry biomass, with a mean value of 19.97 g/pot, representing a percentage increase of 53.61 compared to the control. Similarly, the utilization of 1 mM GABA resulted in a mean root dry biomass of 15.89 g/pot, indicating a percentage increase of 22.26. When ectoine (ECT) was used as the osmoprotectant in the absence of AMF, the control group (0 mM ECT) exhibited a mean root dry biomass of 12.99 g/pot. However, the inclusion of 0.25 mM ECT led to a significant increase in root dry biomass, with a mean value of 17.64 g/pot, signifying a percentage increase of 35.79. Furthermore, the use of 0.50 mM ECT resulted in a mean root dry biomass of 20.33 g/pot, reflecting a percentage increase of 56.49 compared to the control. With AMF, the control group (0 mM GABA) displayed a mean root dry biomass of 14.65 g/pot. The introduction of 0.5 mM GABA significantly increased the root dry biomass to 21.30 g/pot, representing a percentage increase of 45.37. Similarly, the utilization of 1 mM GABA yielded a mean root dry biomass of 17.93 g/pot, indicating a percentage increase of 22.33 compared to the control. Under the presence of AMF and without the addition of ECT, the mean root dry biomass was 15.21 g/pot. However, the inclusion of 0.25 mM ECT led to a significant increase in root dry biomass, with a mean value of 19.63 g/pot, signifying a percentage increase of 29.04. Furthermore, the use of 0.50 mM ECT resulted in a mean root dry biomass of 20.91 g/pot, representing a percentage increase of 37.44 compared to the control (Fig. 3B).

### Leaf potassium

**Fig. 4:**
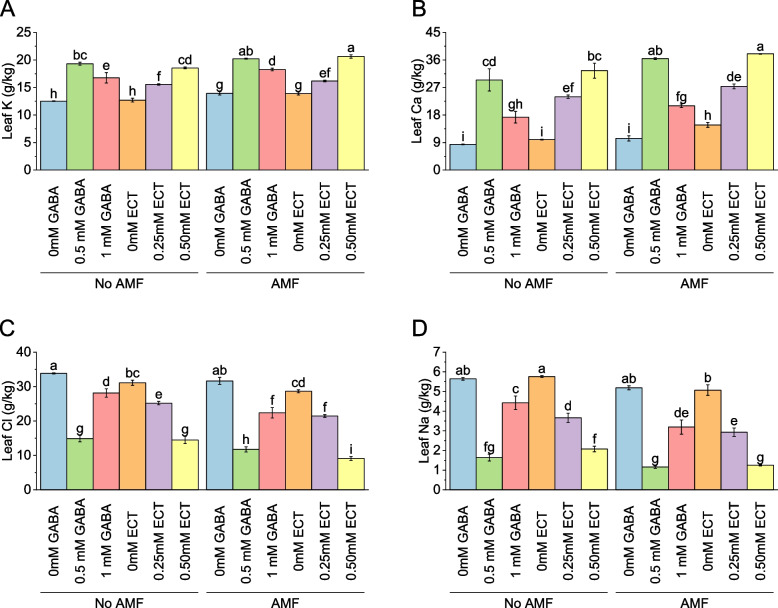
Effect of osmoprotectants γ-Aminobutyric acid (GABA) and ectoine (ECT) different foliar application rates with and with AMF on leaf K (**A**), leaf Ca (**B**), leaf Cl (**C**) and leaf Na (**D**) of cotton cultivated in salinity stress (soil EC = 5.64 dS/m). Bars are means of 3 replicates ± SE. Different letters on bars showed significant changes at p ≤ 0.05; Fisher’s LSD

When AMF was absent, the leaf K content varied between 12.52 g/kg and 18.55 g/kg. The addition of 0.5 mM GABA resulted in a significant increase of 54.41% in leaf K content compared to the absence of AMF. Similarly, the addition of 1 mM GABA led to a 33.96% increase in leaf K content. For ECT treatments without AMF, the leaf K content ranged from 12.71 g/kg to 18.55 g/kg. The addition of 0.5 mM ECT resulted in a significant increase of 45.98% in leaf K content compared to the absence of AMF. In the presence of AMF, the leaf K content varied between 13.94 g/kg and 20.65 g/kg. The addition of 0.5 mM GABA led to a 45.02% increase in leaf K content compared to AMF presence without GABA. Similarly, the addition of 1 mM GABA resulted in a 31.00% increase in leaf K content. For ECT treatments with AMF, the leaf K content ranged from 13.90 g/kg to 20.65 g/kg. The addition of 0.50 mM ECT led to a significant increase of 48.51% in leaf K content compared to AMF presence without ECT (Fig. 4A).

### Leaf calcium

Without AMF, the leaf Ca content varied between 8.40 g/kg and 32.50 g/kg. The addition of 0.5 mM GABA resulted in a significant increase of 250.94% in leaf Ca content compared to the absence of AMF. Similarly, the addition of 1 mM GABA led to a 105.70% increase in leaf Ca content. For ECT treatments without AMF, the leaf Ca content ranged from 9.98 g/kg to 32.50 g/kg. The addition of 0.25 mM ECT resulted in a significant increase of 140.13% in leaf Ca content compared to the absence of AMF. In AMF, the leaf Ca content varied between 10.36 g/kg and 38.00 g/kg. The addition of 0.5 mM GABA led to a 251.59% increase in leaf Ca content compared to AMF presence without GABA. Similarly, the addition of 1 mM GABA resulted in a 102.82% increase in leaf Ca content. For ECT treatments with AMF, the leaf Ca content ranged from 14.74 g/kg to 38.00 g/kg. The addition of 0.50 mM ECT led to a significant increase of 157.75% in leaf Ca content compared to AMF presence without ECT (Fig. 4B).

### Leaf chloride

In case of no AMF, the leaf Cl content ranged from 33.86 g/kg to 14.48 g/kg. The addition of 0.5 mM GABA resulted in a significant decrease of -56.07% in leaf Cl content compared to the absence of AMF. Similarly, the addition of 1 mM GABA led to a -16.86% decrease in leaf Cl content. For ECT treatments without AMF, the leaf Cl content varied from 31.12 g/kg to 14.48 g/kg. The addition of 0.25 mM ECT resulted in a significant decrease of -19.05% in leaf Cl content compared to the absence of AMF. In the presence of AMF, the leaf Cl content ranged from 31.67 g/kg to 9.10 g/kg. The addition of 0.5 mM GABA led to a significant decrease of -62.90% in leaf Cl content compared to AMF presence without GABA. Similarly, the addition of 1 mM GABA resulted in a -29.34% decrease in leaf Cl content. For ECT treatments with AMF, the leaf Cl content ranged from 28.65 g/kg to 9.10 g/kg. The addition of 0.50 mM ECT resulted in a significant decrease of -68.24% in leaf Cl content compared to AMF presence without ECT (Fig. 4C).

### Leaf sodium

In the absence of AMF, the leaf Na content ranged from 5.65 g/kg to 2.07 g/kg. The addition of 0.5 mM GABA resulted in a significant decrease of -70.96% in leaf Na content compared to the absence of AMF. Similarly, the addition of 1 mM GABA led to a -21.60% decrease in leaf Na content. For ECT treatments without AMF, the leaf Na content varied from 5.77 g/kg to 2.07 g/kg. The addition of 0.25 mM ECT resulted in a significant decrease of -36.42% in leaf Na content compared to the absence of AMF. Under AMF, the leaf Na content ranged from 5.19 g/kg to 1.25 g/kg. The addition of 0.5 mM GABA led to a significant decrease of -77.71% in leaf Na content compared to AMF presence without GABA. Similarly, the addition of 1 mM GABA resulted in a -38.51% decrease in leaf Na content. For ECT treatments with AMF, the leaf Na content ranged from 5.07 g/kg to 1.25 g/kg. The addition of 0.50 mM ECT resulted in a significant decrease of -75.29% in leaf Na content compared to AMF presence without ECT (Fig. 4D).

### Stem potassium

**Fig. 5:**
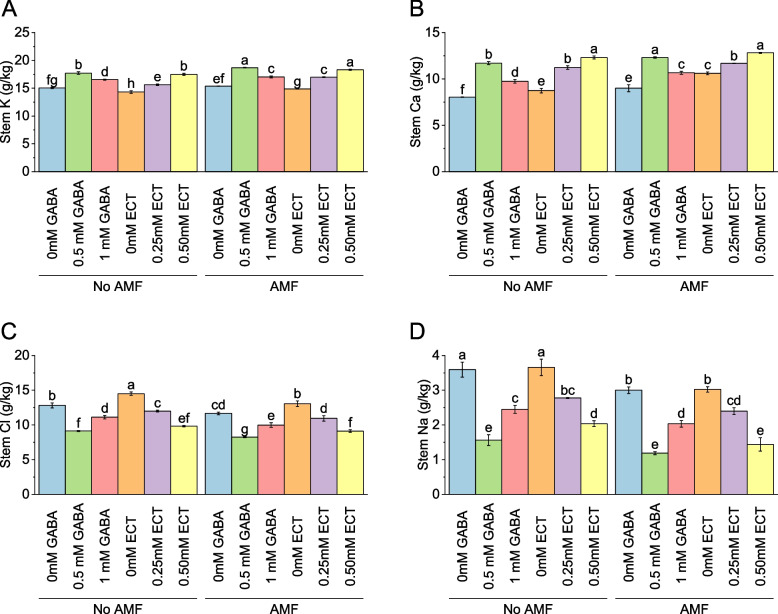
Effect of osmoprotectants γ-Aminobutyric acid (GABA) and ectoine (ECT) different foliar application rates with and with AMF on stem K (**A**), stem Ca (**B**), stem Cl (**C**) and stem Na (**D**) of cotton cultivated in salinity stress (soil EC = 5.64 dS/m). Bars are means of 3 replicates ± SE. Different letters on bars showed significant changes at p ≤ 0.05; Fisher’s LSD

The stem K content ranged from 15.06 g/kg to 17.50 g/kg in the absence of AMF. The addition of 0.5 mM GABA resulted in a 17.67% increase in stem K content compared to the absence of AMF. Similarly, the addition of 1 mM GABA led to a 9.91% increase in stem K content. For ECT treatments without AMF, the stem K content varied from 14.32 g/kg to 17.50 g/kg. The addition of 0.50 mM ECT resulted in a 22.16% increase in stem K content compared to the absence of AMF. When AMF was applied, the stem K content ranged from 15.36 g/kg to 18.32 g/kg. The addition of 0.5 mM GABA led to a 21.73% increase in stem K content compared to AMF presence without GABA. Similarly, the addition of 1 mM GABA resulted in a 10.94% increase in stem K content. For ECT treatments with AMF, the stem K content ranged from 14.88 g/kg to 18.32 g/kg. The addition of 0.50 mM ECT resulted in a 23.09% increase in stem K content compared to AMF presence without ECT (Fig. 5A).

### Stem calcium

In the absence of AMF, applying 0 mM GABA resulted in an average stem calcium level of 8.04 g/kg. When 0.5 mM GABA was introduced, the stem calcium level increased to 11.72 g/kg, representing a percentage increase of 45.74% compared to the control. Applying 1 mM GABA resulted in a mean stem calcium level of 9.74 g/kg, corresponding to a percentage increase of 21.18% compared to the control. Similarly, in the presence of AMF, with 0 mM GABA, the average stem calcium level was 9.00 g/kg. Applying 0.5 mM GABA led to an increase in stem calcium levels, with an average of 12.31 g/kg, representing a percentage increase of 36.78% compared to the control. The application of 1 mM GABA resulted in an average stem calcium level of 10.66 g/kg, corresponding to a percentage increase of 18.47% compared to the control. For the treatments with ECT, in the absence of AMF, applying 0 mM ECT resulted in a mean stem calcium level of 8.74 g/kg. Introducing 0.25 mM ECT caused an increase to 11.23 g/kg, corresponding to a percentage increase of 28.39% compared to the control. Applying 0.50 mM ECT resulted in an average stem calcium level of 12.31 g/kg, representing a percentage increase of 40.83% compared to the control. When AMF was present, the average stem calcium level with 0 mM ECT was 10.60 g/kg. Introducing 0.25 mM ECT caused a slight increase to 11.69 g/kg, representing a percentage increase of 10.23% compared to the control. Applying 0.50 mM ECT resulted in an average stem calcium level of 12.82 g/kg, corresponding to a percentage increase of 20.93% compared to the control (Fig. 5B).

### Stem chloride

Application of no AMF and 0 mM GABA resulted in an average stem chloride level of 12.82 g/kg. When 0.5 mM GABA was introduced, the stem chloride level decreased to 9.14 g/kg, representing a percentage decrease of -28.73% compared to the control. Applying 1 mM GABA resulted in a mean stem chloride level of 11.13 g/kg, corresponding to a percentage decrease of -13.21% compared to the control. Under 0 mM GABA + AMF, the average stem chloride level was 11.67 g/kg. Applying 0.5 mM GABA led to a decrease in stem chloride levels, with an average of 8.26 g/kg, representing a percentage decrease of -29.20% compared to the control. The application of 1 mM GABA resulted in an average stem chloride level of 9.99 g/kg, corresponding to a percentage decrease of -14.40% compared to the control. In no AMF, applying 0 mM ECT resulted in a mean stem chloride level of 14.51 g/kg. Introducing 0.25 mM ECT caused a decrease to 12.00 g/kg, corresponding to a percentage decrease of -17.28% compared to the control. Applying 0.50 mM ECT resulted in an average stem chloride level of 9.82 g/kg, representing a percentage decrease of -32.32% compared to the control. When AMF was present, the average stem chloride level with 0 mM ECT was 13.06 g/kg. Introducing 0.25 mM ECT caused a decrease to 10.96 g/kg, representing a percentage decrease of -16.10% compared to the control. Applying 0.50 mM ECT resulted in an average stem chloride level of 9.12 g/kg, corresponding to a percentage decrease of -30.12% compared to the control (Fig. 5C).

### Stem sodium

In the absence of AMF, the stem Na content ranged from 3.59 g/kg to 2.04 g/kg. The addition of 0.5 mM GABA resulted in a significant decrease of -56.44% in stem Na content compared to the absence of AMF. Similarly, the addition of 1 mM GABA led to a -31.76% decrease in stem Na content. For ECT treatments without AMF, the stem Na content varied from 3.66 g/kg to 2.04 g/kg. The addition of 0.25 mM ECT resulted in a significant decrease of -24.10% in stem Na content compared to the absence of AMF. For AMF, the stem Na content ranged from 3.00 g/kg to 1.44 g/kg. The addition of 0.5 mM GABA led to a significant decrease of -60.35% in stem Na content compared to AMF presence without GABA. Similarly, the addition of 1 mM GABA resulted in a -32.19% decrease in stem Na content. For ECT treatments with AMF, the stem Na content ranged from 3.02 g/kg to 1.44 g/kg. The addition of 0.50 mM ECT resulted in a significant decrease of -52.42% in stem Na content compared to AMF presence without ECT (Fig. 5D).

### Bur potassium

**Fig. 6:**
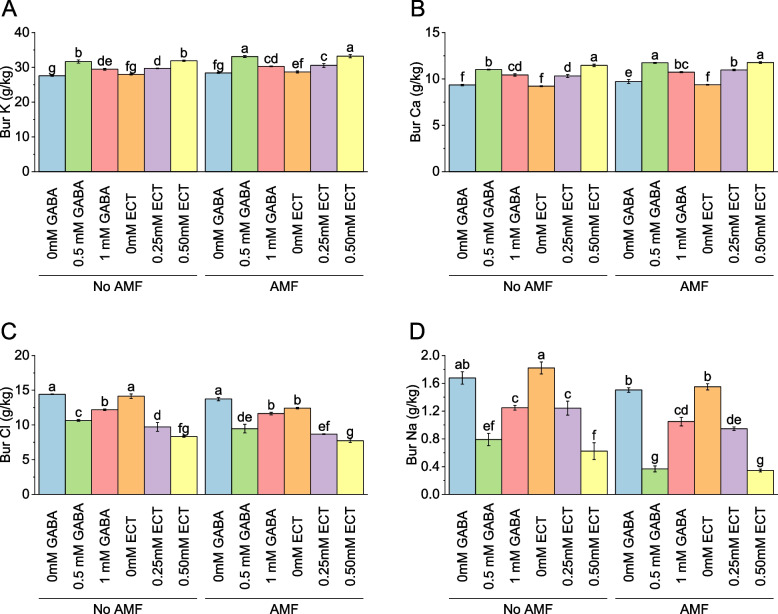
Effect of osmoprotectants γ-Aminobutyric acid (GABA) and ectoine (ECT) different foliar application rates with and with AMF on bur K (**A**), bur Ca (**B**), bur Cl (**C**) and bur Na (**D**) of cotton cultivated in salinity stress (soil EC = 5.64 dS/m). Bars are means of 3 replicates ± SE. Different letters on bars showed significant changes at p ≤ 0.05; Fisher’s LSD

In the absence of AMF, the mean bur K value was 27.59 g/kg for plants treated with 0 mM GABA. When the concentration of GABA increased to 0.5 mM, the mean bur K value rose to 31.68 g/kg, representing a percentage change of 14.86%. Similarly, with 1 mM GABA, the mean bur K value was 29.47 g/kg, reflecting a percentage change of 6.82%. For plants treated with ECT as the osmoprotectant, the mean bur K value without AMF was 27.99 g/kg for 0 mM ECT. When the concentration of ECT increased to 0.25 mM and 0.50 mM, the mean bur K values were 29.69 g/kg and 31.89 g/kg, respectively, with corresponding percentage changes of 6.05% and 13.94%. When AMF were present, the mean bur K value for plants treated with 0 mM GABA increased to 28.41 g/kg. Introducing 0.5 mM GABA resulted in a mean bur K value of 33.12 g/kg, reflecting a percentage change of 16.58%. With 1 mM GABA, the mean bur K value was 30.30 g/kg, representing a percentage change of 6.65%. For plants treated with 0 mM ECT in the presence of AMF, the mean bur K value was 28.70 g/kg. When the concentration of ECT increased to 0.25 mM and 0.50 mM, the mean bur K values were 30.56 g/kg and 33.24 g/kg, respectively, with corresponding percentage changes of 6.47% and 15.84% (Fig. 6A).

### Bur calcium

Without AMF, the mean bur calcium value was 9.35 g/kg for plants treated with 0 mM GABA. When the concentration of GABA increased to 0.5 mM, the mean bur calcium value rose to 11.01 g/kg, representing a percentage change of 17.79%. Similarly, with 1 mM GABA, the mean bur calcium value was 10.43 g/kg, reflecting a percentage change of 11.56%. For plants treated with ECT as the osmoprotectant, the mean bur calcium value without AMF was 9.22 g/kg for 0 mM ECT. When the concentration of ECT increased to 0.25 mM and 0.50 mM, the mean bur calcium values were 10.32 g/kg and 11.47 g/kg, respectively, with corresponding percentage changes of 11.95% and 24.42%. In case of AMF were present, the mean bur calcium value for plants treated with 0 mM GABA increased to 9.72 g/kg. Introducing 0.5 mM GABA resulted in a mean bur calcium value of 11.74 g/kg, reflecting a percentage change of 20.77%. With 1 mM GABA, the mean bur calcium value was 10.73 g/kg, representing a percentage change of 10.41%. For plants treated with 0 mM ECT in the presence of AMF, the mean bur calcium value was 9.38 g/kg. When the concentration of ECT increased to 0.25 mM and 0.50 mM, the mean bur calcium values were 10.97 g/kg and 11.77 g/kg, respectively, with corresponding percentage changes of 16.98% and 25.56% (Fig. 6B).

### Bur chloride

Under no AMF, the mean bur chloride value was 14.43 g/kg for plants treated with 0 mM GABA. When the concentration of GABA increased to 0.5 mM, the mean bur chloride value decreased to 10.65 g/kg, representing a percentage change of -26.18%. Similarly, with 1 mM GABA, the mean bur chloride value was 12.20 g/kg, reflecting a percentage change of -15.43%. For plants treated with ECT as the osmoprotectant, the mean bur chloride value without AMF was 14.15 g/kg for 0 mM ECT. When the concentration of ECT increased to 0.25 mM and 0.50 mM, the mean bur chloride values were 9.72 g/kg and 8.34 g/kg, respectively, with corresponding percentage changes of -31.27% and -41.07%. In AMF were present, the mean bur chloride value for plants treated with 0 mM GABA decreased to 13.73 g/kg. Introducing 0.5 mM GABA resulted in a mean bur chloride value of 9.47 g/kg, reflecting a percentage change of -31.05%. With 1 mM GABA, the mean bur chloride value was 11.64 g/kg, representing a percentage change of -15.27%. For plants treated with 0 mM ECT in the presence of AMF, the mean bur chloride value was 12.43 g/kg. When the concentration of ECT increased to 0.25 mM and 0.50 mM, the mean bur chloride values were 8.68 g/kg and 7.74 g/kg, respectively, with corresponding percentage changes of -30.16% and -37.74% (Figu. 6C).

### Bur sodium

In the absence of AMF, the mean bur sodium value was 1.68 g/kg for plants treated with 0 mM GABA. When the concentration of GABA increased to 0.5 mM, the mean bur sodium value decreased to 0.79 g/kg, representing a percentage change of -52.84%. Similarly, with 1 mM GABA, the mean bur sodium value was 1.25 g/kg, reflecting a percentage change of -25.66%. For plants treated with ECT as the osmoprotectant, the mean bur sodium value without AMF was 1.82 g/kg for 0 mM ECT. When the concentration of ECT increased to 0.25 mM and 0.50 mM, the mean bur sodium values were 1.24 g/kg and 0.62 g/kg, respectively, with corresponding percentage changes of -31.76% and -65.73%. When AMF were present, the mean bur sodium value for plants treated with 0 mM GABA decreased to 1.50 g/kg. Introducing 0.5 mM GABA resulted in a mean bur sodium value of 0.37 g/kg, reflecting a percentage change of -75.61%. With 1 mM GABA, the mean bur sodium value was 1.05 g/kg, representing a percentage change of -30.24%. For plants treated with 0 mM ECT in the presence of AMF, the mean bur sodium value was 1.55 g/kg. When the concentration of ECT increased to 0.25 mM and 0.50 mM, the mean bur sodium values were 0.95 g/kg and 0.35 g/kg, respectively, with corresponding percentage changes of -38.90% and -77.71% (Fig. 6D).

### Seed potassium

**Fig. 7:**
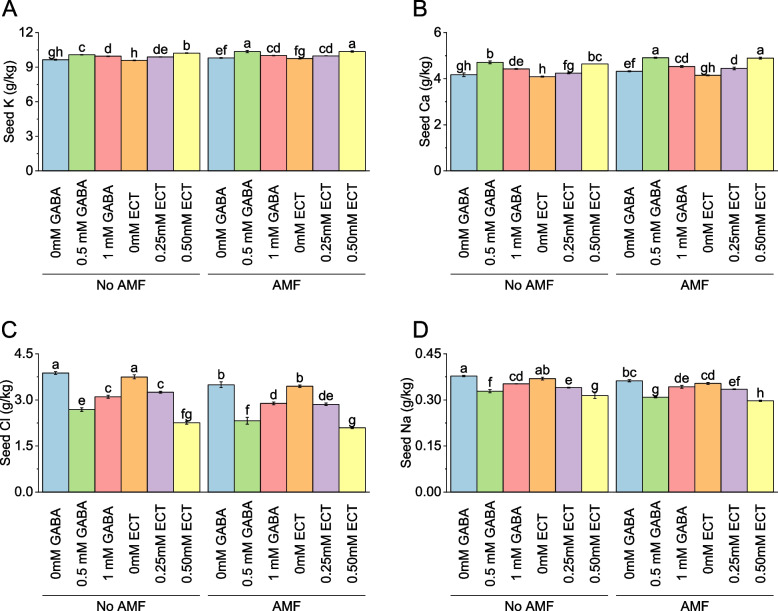
Effect of osmoprotectants γ-Aminobutyric acid (GABA) and ectoine (ECT) different foliar application rates with and with AMF on seed K (**A**), seed Ca (**B**), seed Cl (**C**) and seed Na (**D**) of cotton cultivated in salinity stress (soil EC = 5.64 dS/m). Bars are means of 3 replicates ± SE. Different letters on bars showed significant changes at p ≤ 0.05; Fisher’s LSD

At no AMF, the mean seed potassium value was 9.65 g/kg for seeds treated with 0 mM GABA. When the concentration of GABA increased to 0.5 mM, the mean seed potassium value rose to 10.08 g/kg, representing a percentage change of 4.49%. Similarly, with 1 mM GABA, the mean seed potassium value was 9.95 g/kg, reflecting a percentage change of 3.11%. For seeds treated with ECT as the osmoprotectant, the mean seed potassium value without AMF was 9.59 g/kg for 0 mM ECT. When the concentration of ECT increased to 0.25 mM and 0.50 mM, the mean seed potassium values were 9.89 g/kg and 10.23 g/kg, respectively, with corresponding percentage changes of 3.18% and 6.68%. When AMF were present, the mean seed potassium value for seeds treated with 0 mM GABA increased to 9.80 g/kg. Introducing 0.5 mM GABA resulted in a mean seed potassium value of 10.35 g/kg, reflecting a percentage change of 5.61%. With 1 mM GABA, the mean seed potassium value was 10.01 g/kg, representing a percentage change of 2.14%. For seeds treated with 0 mM ECT in the presence of AMF, the mean seed potassium value was 9.75 g/kg. When the concentration of ECT increased to 0.25 mM and 0.50 mM, the mean seed potassium values were 9.98 g/kg and 10.36 g/kg, respectively, with corresponding percentage changes of 2.35% and 6.31% (Fig. 7A).

### Seed calcium

For no AMF, the mean seed calcium value was 4.17 g/kg for seeds treated with 0 mM GABA. When the concentration of GABA increased to 0.5 mM, the mean seed calcium value rose to 4.71 g/kg, representing a percentage change of 12.87%. Similarly, with 1 mM GABA, the mean seed calcium value was 4.42 g/kg, reflecting a percentage change of 6.01%. For seeds treated with ECT as the osmoprotectant, the mean seed calcium value without AMF was 4.09 g/kg for 0 mM ECT. When the concentration of ECT increased to 0.25 mM and 0.50 mM, the mean seed calcium values were 4.24 g/kg and 4.64 g/kg, respectively, with corresponding percentage changes of 3.69% and 13.55%. Under AMF, the mean seed calcium value for seeds treated with 0 mM GABA increased to 4.32 g/kg. Introducing 0.5 mM GABA resulted in a mean seed calcium value of 4.91 g/kg, reflecting a percentage change of 13.51%. With 1 mM GABA, the mean seed calcium value was 4.53 g/kg, representing a percentage change of 4.81%. For seeds treated with 0 mM ECT in the presence of AMF, the mean seed calcium value was 4.15 g/kg. When the concentration of ECT increased to 0.25 mM and 0.50 mM, the mean seed calcium values were 4.45 g/kg and 4.89 g/kg, respectively, with corresponding percentage changes of 7.25% and 18.01% (Fig. 7B).

### Seed chloride

In the absence of AMF, the mean seed chloride value was 3.88 g/kg for seeds treated with 0 mM GABA. When the concentration of GABA increased to 0.5 mM, the mean seed chloride value decreased to 2.69 g/kg, representing a percentage change of -30.70%. Similarly, with 1 mM GABA, the mean seed chloride value was 3.10 g/kg, reflecting a percentage change of -19.94%. For seeds treated with ECT as the osmoprotectant, the mean seed chloride value without AMF was 3.75 g/kg for 0 mM ECT. When the concentration of ECT increased to 0.25 mM and 0.50 mM, the mean seed chloride values were 3.25 g/kg and 2.25 g/kg, respectively, with corresponding percentage changes of -13.25% and -39.88%. When AMF were present, the mean seed chloride value for seeds treated with 0 mM GABA decreased to 3.49 g/kg. Introducing 0.5 mM GABA resulted in a mean seed chloride value of 2.32 g/kg, reflecting a percentage change of -33.52%. With 1 mM GABA, the mean seed chloride value was 2.89 g/kg, representing a percentage change of -17.34%. For seeds treated with 0 mM ECT in the presence of AMF, the mean seed chloride value was 3.45 g/kg. When the concentration of ECT increased to 0.25 mM and 0.50 mM, the mean seed chloride values were 2.86 g/kg and 2.09 g/kg, respectively, with corresponding percentage changes of -17.05% and -39.32% (Fig. 7C).

### Seed sodium

In case of no AMF, the mean seed sodium value was 0.38 g/kg for seeds treated with 0 mM GABA. When the concentration of GABA increased to 0.5 mM, the mean seed sodium value decreased to 0.33 g/kg, representing a percentage change of -13.13%. Similarly, with 1 mM GABA, the mean seed sodium value was 0.35 g/kg, reflecting a percentage change of -6.71%. For seeds treated with ECT as the osmoprotectant, the mean seed sodium value without AMF was 0.37 g/kg for 0 mM ECT. When the concentration of ECT increased to 0.25 mM and 0.50 mM, the mean seed sodium values were 0.34 g/kg and 0.31 g/kg, respectively, with corresponding percentage changes of -7.90% and -14.98%. Applying AMF with 0 mM GABA, the mean seed sodium value for seeds treated decreased to 0.36 g/kg. Introducing 0.5 mM GABA resulted in a mean seed sodium value of 0.31 g/kg, reflecting a percentage change of -14.75%. With 1 mM GABA, the mean seed sodium value was 0.34 g/kg, representing a percentage change of -5.51%. For seeds treated with 0 mM ECT in the presence of AMF, the mean seed sodium value was 0.35 g/kg. When the concentration of ECT increased to 0.25 mM and 0.50 mM, the mean seed sodium values were 0.33 g/kg and 0.30 g/kg, respectively, with corresponding percentage changes of -5.33% and -16.04% (Fig. 7D).

### Chlorophyll a

**Fig. 8:**
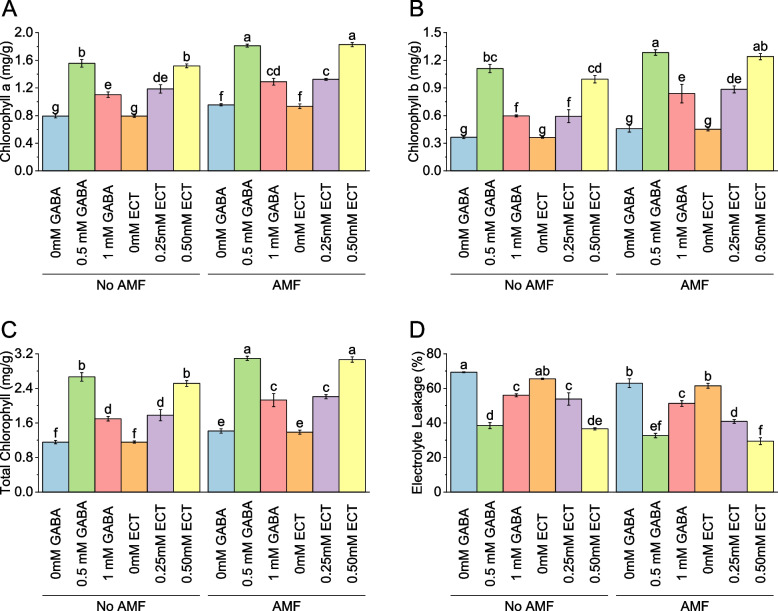
Effect of osmoprotectants γ-Aminobutyric acid (GABA) and ectoine (ECT) different foliar application rates with and with AMF on chlorophyll a (**A**), chlorophyll b (**B**), total chlorophyll (**C**) and electrolyte leakage (**D**) of cotton cultivated in salinity stress (soil EC = 5.64 dS/m). Bars are means of 3 replicates ± SE. Different letters on bars showed significant changes at p ≤ 0.05; Fisher’s LSD

In the absence of AMF, the mean chlorophyll a value was 0.79 mg/g for samples treated with 0 mM GABA. When the concentration of GABA increased to 0.5 mM, the mean chlorophyll a value rose to 1.56 mg/g, representing a percentage change of 96.25%. Similarly, with 1 mM GABA, the mean chlorophyll a value was 1.10 mg/g, reflecting a percentage change of 39.15%. For samples treated with ECT as the osmoprotectant, the mean chlorophyll a value without AMF was 0.79 mg/g for 0 mM ECT. When the concentration of ECT increased to 0.25 mM and 0.50 mM, the mean chlorophyll a values were 1.19 mg/g and 1.52 mg/g, respectively, with corresponding percentage changes of 49.61% and 91.66%. When AMF were present, the mean chlorophyll a value for samples treated with 0 mM GABA increased to 0.95 mg/g. Introducing 0.5 mM GABA resulted in a mean chlorophyll a value of 1.81 mg/g, reflecting a percentage change of 89.69%. With 1 mM GABA, the mean chlorophyll a value was 1.29 mg/g, representing a percentage change of 35.13%. For samples treated with 0 mM ECT in the presence of AMF, the mean chlorophyll a value was 0.93 mg/g. When the concentration of ECT increased to 0.25 mM and 0.50 mM, the mean chlorophyll a values were 1.32 mg/g and 1.83 mg/g, respectively, with corresponding percentage changes of 41.68% and 95.37% (Fig. 8A).

### Chlorophyll b

For no AMF, the mean chlorophyll b value was 0.36 mg/g for samples treated with 0 mM GABA. When the concentration of GABA increased to 0.5 mM, the mean chlorophyll b value rose to 1.11 mg/g, representing a percentage change of 205.31%. Similarly, with 1 mM GABA, the mean chlorophyll b value was 0.60 mg/g, reflecting a percentage change of 63.81%. For samples treated with ECT as the osmoprotectant, the mean chlorophyll b value without AMF was 0.36 mg/g for 0 mM ECT. When the concentration of ECT increased to 0.25 mM and 0.50 mM, the mean chlorophyll b values were 0.59 mg/g and 1.00 mg/g, respectively, with corresponding percentage changes of 63.22% and 173.82%. In the presence of AMF, the mean chlorophyll b value for samples treated with 0 mM GABA increased to 0.46 mg/g. Introducing 0.5 mM GABA resulted in a mean chlorophyll b value of 1.28 mg/g, reflecting a percentage change of 180.08%. With 1 mM GABA, the mean chlorophyll b value was 0.84 mg/g, representing a percentage change of 82.95%. For samples treated with 0 mM ECT in the presence of AMF, the mean chlorophyll b value was 0.45 mg/g. When the concentration of ECT increased to 0.25 mM and 0.50 mM, the mean chlorophyll b values were 0.88 mg/g and 1.24 mg/g, respectively, with corresponding percentage changes of 95.76% and 174.52% (Fig. 8B).

The mean total chlorophyll value was 1.16 mg/g for samples treated with no AMF and 0 mM GABA. When the concentration of GABA increased to 0.5 mM, the mean total chlorophyll value rose to 2.67 mg/g, representing a percentage change of 130.56%. Similarly, with 1 mM GABA, the mean total chlorophyll value was 1.70 mg/g, reflecting a percentage change of 46.90%. For samples treated with ECT as the osmoprotectant, the mean total chlorophyll value without AMF was 1.16 mg/g for 0 mM ECT. When the concentration of ECT increased to 0.25 mM and 0.50 mM, the mean total chlorophyll values were 1.78 mg/g and 2.51 mg/g, respectively, with corresponding percentage changes of 53.89% and 117.49%. Applying AMF with 0 mM GABA, the mean total chlorophyll value for samples increased to 1.41 mg/g. Introducing 0.5 mM GABA resulted in a mean total chlorophyll value of 3.10 mg/g, reflecting a percentage change of 119.00%. With 1 mM GABA, the mean total chlorophyll value was 2.13 mg/g, representing a percentage change of 50.64%. For samples treated with 0 mM ECT in the presence of AMF, the mean total chlorophyll value was 1.39 mg/g. When the concentration of ECT increased to 0.25 mM and 0.50 mM, the mean total chlorophyll values were 2.21 mg/g and 3.07 mg/g, respectively, with corresponding percentage changes of 59.30% and 121.16% (Fig. 8C).

The results revealed interesting insights into the role of AMF in exacerbating electrolyte leakage in certain treatments compared to those without AMF. When AMF was present alongside 0 mM GABA, the electrolyte leakage increased by approximately 8.85% compared to the treatment without AMF. Similarly, with 0.5 mM GABA, the increase was approximately 15.21%, and with 1 mM GABA, it was approximately 8.30%. Additionally, when 0 mM ECT was combined with AMF, the electrolyte leakage increased by around 6.01%. Notably, the presence of AMF had a more pronounced effect when paired with higher ECT concentrations, showing an increase of approximately 24.43% with 0.25 mM ECT and 19.44% with 0.50 mM ECT, both compared to the corresponding treatments without AMF (Fig. 8D).

### Photosynthetic rate

**Fig. 9:**
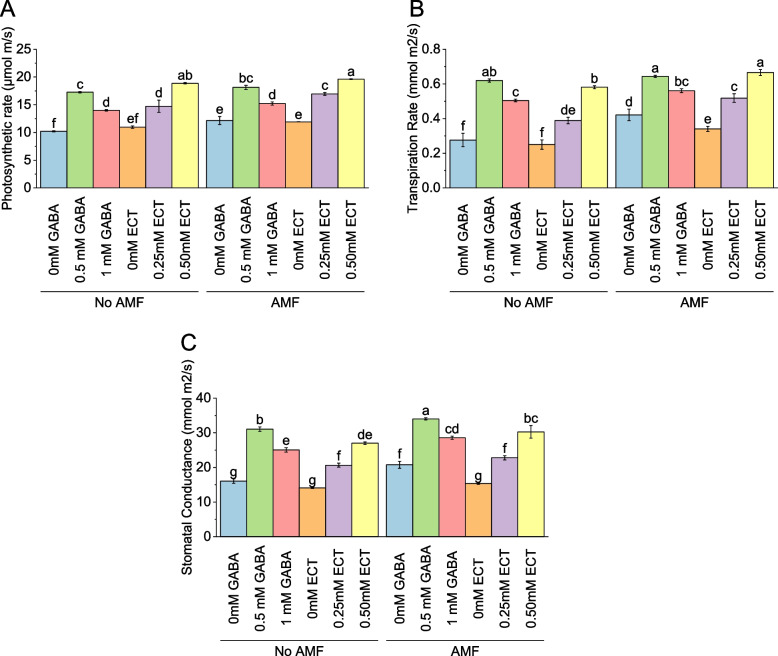
Effect of osmoprotectants γ-Aminobutyric acid (GABA) and ectoine (ECT) different foliar application rates with and with AMF on photosynthetic rate (**A**), transpiration rate (**B**) and stomatal conductance (**C**) of cotton cultivated in salinity stress (soil EC = 5.64 dS/m). Bars are means of 3 replicates ± SE. Different letters on bars showed significant changes at p ≤ 0.05; Fisher’s LSD

In the absence of AMF, the mean photosynthetic rate was 10.21 μmol m^–2^ s^–1^ for samples treated with 0 mM GABA. When the concentration of GABA increased to 0.5 mM, the mean photosynthetic rate rose to 17.26 μmol m^–2^ s^–1^, representing a percentage change of 69.02%. Similarly, with 1 mM GABA, the mean photosynthetic rate was 13.99 μmol m^–2^ s^–1^, reflecting a percentage change of 36.97%. For samples treated with ECT as the osmoprotectant, the mean photosynthetic rate without AMF was 10.98 μmol m^–2^ s^–1^ for 0 mM ECT. When the concentration of ECT increased to 0.25 mM and 0.50 mM, the mean photosynthetic rates were 14.73 μmol m^–2^ s^–1^ and 18.88 μmol m^–2^ s^–1^, respectively, with corresponding percentage changes of 34.19% and 71.97%. When AMF were present, the mean photosynthetic rate for samples treated with 0 mM GABA increased to 12.16 μmol m^–2^ s^–1^. Introducing 0.5 mM GABA resulted in a mean photosynthetic rate of 18.12 μmol m^–2^ s^–1^, reflecting a percentage change of 49.01%. With 1 mM GABA, the mean photosynthetic rate was 15.22 μmol m^–2^ s^–1^, representing a percentage change of 25.14%. For samples treated with 0 mM ECT in the presence of AMF, the mean photosynthetic rate was 11.91 μmol m^–2^ s^–1^. When the concentration of ECT increased to 0.25 mM and 0.50 mM, the mean photosynthetic rates were 16.94 μmol m^–2^ s^–1^ and 19.61 μmol m^–2^ s^–1^, respectively, with corresponding percentage changes of 42.20% and 64.66% (Fig. 9A).

### Transpiration rate

At no AMF, the mean transpiration rate was 0.28 mmol m^−2^ s^−1^ for samples treated with 0 mM GABA. When the concentration of GABA increased to 0.5 mM, the mean transpiration rate rose to 0.62 mmol m^–2^ s^–1^, representing a percentage change of 124.25%. Similarly, with 1 mM GABA, the mean transpiration rate was 0.50 mmol m^–2^ s^–1^, reflecting a percentage change of 82.60%. For samples treated with ECT as the osmoprotectant, the mean transpiration rate without AMF was 0.25 mmol m^–2^ s^–1^ for 0 mM ECT. When the concentration of ECT increased to 0.25 mM and 0.50 mM, the mean transpiration rates were 0.39 mmol m^–2^ s^–1^ and 0.58 mmol m^–2^ s^–1^, respectively, with corresponding percentage changes of 55.70% and 132.39%. Inoculation of AMF, the mean transpiration rate for samples treated with 0 mM GABA increased to 0.42 mmol m^–2^ s^–1^. Introducing 0.5 mM GABA resulted in a mean transpiration rate of 0.64 mmol m^–2^ s^–1^, reflecting a percentage change of 52.74%. With 1 mM GABA, the mean transpiration rate was 0.56 mmol m^–2^ s^–1^, representing a percentage change of 33.20%. For samples treated with 0 mM ECT in the presence of AMF, the mean transpiration rate was 0.34 mmol m^–2^ s^–1^. When the concentration of ECT increased to 0.25 mM and 0.50 mM, the mean transpiration rates were 0.52 mmol m^–2^ s^–1^ and 0.67 mmol m^–2^ s^–1^, respectively, with corresponding percentage changes of 52.19% and 95.40% (Fig. 9B).

### Stomatal conductance

Without AMF, the mean stomatal conductance was 16.04 mmol m^–2^ s^–1^ for samples treated with 0 mM GABA. When the concentration of GABA increased to 0.5 mM, the mean stomatal conductance rose to 31.07 mmol m^–2^ s^–1^, representing a percentage change of 93.70%. Similarly, with 1 mM GABA, the mean stomatal conductance was 25.07 mmol m^–2^ s^–1^, reflecting a percentage change of 56.33%. For samples treated with ECT as the osmoprotectant, the mean stomatal conductance without AMF was 14.09 mmol m^–2^ s^–1^ for 0 mM ECT. When the concentration of ECT increased to 0.25 mM and 0.50 mM, the mean stomatal conductance values were 20.64 mmol m^–2^ s^–1^ and 26.98 mmol m^–2^ s^–1^, respectively, with corresponding percentage changes of 46.47% and 91.51%. In AMF, the mean stomatal conductance for samples treated with 0 mM GABA increased to 20.76 mmol m^–2^ s^–1^. Introducing 0.5 mM GABA resulted in a mean stomatal conductance of 34.02 mmol m^–2^ s^–1^, reflecting a percentage change of 63.92%. With 1 mM GABA, the mean stomatal conductance was 28.57 mmol m^–2^ s^–1^, representing a percentage change of 37.63%. For samples treated with 0 mM ECT in the presence of AMF, the mean stomatal conductance was 15.36 mmol m^–2^ s^–1^. When the concentration of ECT increased to 0.25 mM and 0.50 mM, the mean stomatal conductance values were 22.78 mmol m^–2^ s^–1^ and 30.28 mmol m^–2^ s^–1^, respectively, with corresponding percentage changes of 48.28% and 97.13% (Fig. 9C).

**Fig. 10:**
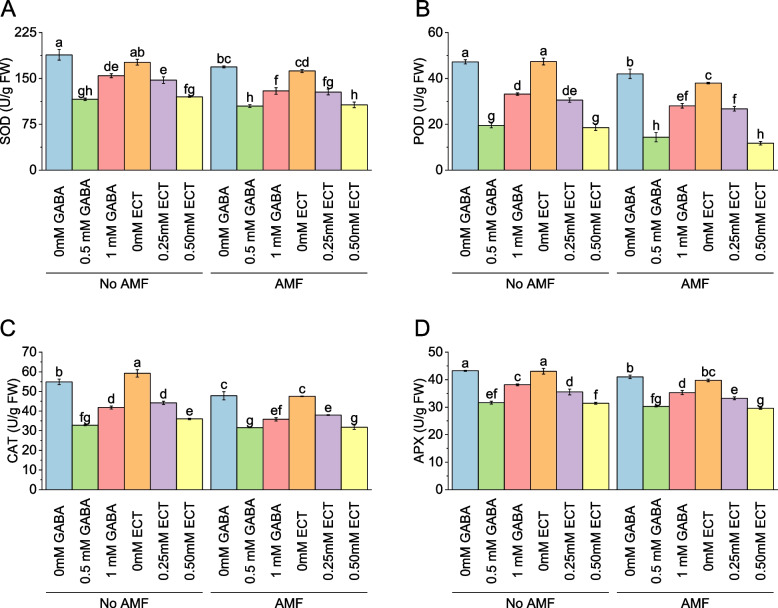
Effect of osmoprotectants γ-Aminobutyric acid (GABA) and ectoine (ECT) different foliar application rates with and with AMF on SOD (**A**), POD (**B**), CAT (**C**) and APX (**D**) of cotton cultivated in salinity stress (soil EC = 5.64 dS/m). Bars are means of 3 replicates ± SE. Different letters on bars showed significant changes at p ≤ 0.05; Fisher’s LSD

In the absence of AMF, the mean SOD activity was 188.59 U/g FW for samples treated with 0 mM GABA. When the concentration of GABA increased to 0.5 mM, the mean SOD activity decreased to 115.94 U/g FW, representing a percentage change of -38.52%. Similarly, with 1 mM GABA, the mean SOD activity decreased to 154.40 U/g FW, reflecting a percentage change of -18.13%. For samples treated with ECT as the osmoprotectant, the mean SOD activity without AMF was 176.44 U/g FW for 0 mM ECT. When the concentration of ECT increased to 0.25 mM and 0.50 mM, the mean SOD activity values were 147.17 U/g FW and 119.92 U/g FW, respectively, with corresponding percentage changes of -16.59% and -32.03%. When AMF were present, the mean SOD activity for samples treated with 0 mM GABA decreased to 168.83 U/g FW. Introducing 0.5 mM GABA resulted in a mean SOD activity of 104.87 U/g FW, reflecting a percentage change of -37.89%. With 1 mM GABA, the mean SOD activity decreased to 129.57 U/g FW, representing a percentage change of -23.25%. For samples treated with 0 mM ECT in the presence of AMF, the mean SOD activity was 162.19 U/g FW. When the concentration of ECT increased to 0.25 mM and 0.50 mM, the mean SOD activity values were 127.83 U/g FW and 106.83 U/g FW, respectively, with corresponding percentage changes of -21.19% and -34.13% (Fig. 10A).

Under no AMF, the mean POD activity was 47.25 U/g FW for samples treated with 0 mM GABA. When the concentration of GABA increased to 0.5 mM, the mean POD activity decreased to 19.49 U/g FW, representing a percentage change of -58.74%. Similarly, with 1 mM GABA, the mean POD activity decreased to 33.20 U/g FW, reflecting a percentage change of -29.73%. For samples treated with ECT as the osmoprotectant, the mean POD activity without AMF was 47.41 U/g FW for 0 mM ECT. When the concentration of ECT increased to 0.25 mM and 0.50 mM, the mean POD activity values were 30.53 U/g FW and 18.58 U/g FW, respectively, with corresponding percentage changes of -35.60% and -60.82%. When AMF were present, the mean POD activity for samples treated with 0 mM GABA decreased to 42.01 U/g FW. Introducing 0.5 mM GABA resulted in a mean POD activity of 14.33 U/g FW, reflecting a percentage change of -65.89%. With 1 mM GABA, the mean POD activity decreased to 28.08 U/g FW, representing a percentage change of -33.17%. For samples treated with 0 mM ECT in the presence of AMF, the mean POD activity was 37.98 U/g FW. When the concentration of ECT increased to 0.25 mM and 0.50 mM, the mean POD activity values were 26.75 U/g FW and 11.77 U/g FW, respectively, with corresponding percentage changes of -29.57% and -69.01% (Fig. 10B).

In the absence of AMF, the mean CAT activity was 54.84 U/g FW for samples treated with 0 mM GABA. When the concentration of GABA increased to 0.5 mM, the mean CAT activity decreased to 32.84 U/g FW, representing a percentage change of -40.12%. Similarly, with 1 mM GABA, the mean CAT activity decreased to 41.81 U/g FW, reflecting a percentage change of -23.75%. For samples treated with ECT as the osmoprotectant, the mean CAT activity without AMF was 59.21 U/g FW for 0 mM ECT. When the concentration of ECT increased to 0.25 mM and 0.50 mM, the mean CAT activity values were 44.23 U/g FW and 36.01 U/g FW, respectively, with corresponding percentage changes of -25.30% and -39.19%. When AMF were present, the mean CAT activity for samples treated with 0 mM GABA decreased to 47.82 U/g FW. Introducing 0.5 mM GABA resulted in a mean CAT activity of 31.58 U/g FW, reflecting a percentage change of -33.95%. With 1 mM GABA, the mean CAT activity decreased to 35.80 U/g FW, representing a percentage change of -25.12%. For samples treated with 0 mM ECT in the presence of AMF, the mean CAT activity was 47.52 U/g FW. When the concentration of ECT increased to 0.25 mM and 0.50 mM, the mean CAT activity values were 37.99 U/g FW and 31.81 U/g FW, respectively, with corresponding percentage changes of -20.05% and -33.07% (Fig. 10C).

In the absence of AMF, the mean Ascorbate Peroxidase (APX) activity was 43.23 U/g FW for samples treated with 0 mM GABA. Increasing the concentration of GABA to 0.5 mM led to a decrease in the mean APX activity to 31.65 U/g FW, resulting in a percentage change of -26.79%. Similarly, when treated with 1 mM GABA, the mean APX activity decreased to 38.15 U/g FW, showing a percentage change of -11.75%. For samples treated with ECT as the osmoprotectant without AMF, the mean APX activity was 43.07 U/g FW for 0 mM ECT. As the concentration of ECT increased to 0.25 mM and 0.50 mM, the mean APX activity values were 35.53 U/g FW and 31.43 U/g FW, respectively, with corresponding percentage changes of -17.51% and -27.02%. With the presence of AMF, the mean APX activity for samples treated with 0 mM GABA decreased to 41.03 U/g FW. Introducing 0.5 mM GABA resulted in a mean APX activity of 30.28 U/g FW, showing a percentage change of -26.20%. With 1 mM GABA, the mean APX activity decreased to 35.30 U/g FW, representing a percentage change of -13.97%. For samples treated with 0 mM ECT in the presence of AMF, the mean APX activity was 39.74 U/g FW. As the concentration of ECT increased to 0.25 mM and 0.50 mM, the mean APX activity values were 33.25 U/g FW and 29.61 U/g FW, respectively, with corresponding percentage changes of -16.33% and -25.49% (Fig. 10D).

**Fig. 11:**
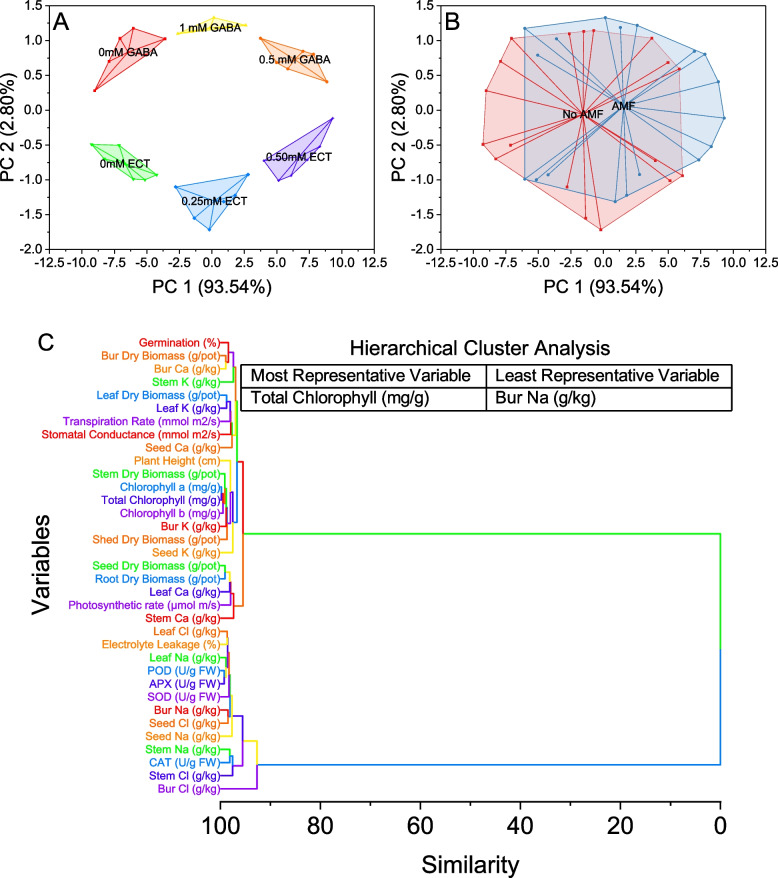
Cluster plot convex hull for treatments (**A**), AMF levels (**B**), and hierarchical cluster plot (**C**) for studied attributes

The provided data represents a cluster plot with two principal components, PC 1 and PC 2, explaining 93.54% and 2.80% of the variance, respectively. Examining the plot, we observe distinct clusters formed by data points associated with different osmoprotectants. The "0 mM GABA" cluster is located in the lower left quadrant, encompassing data points with PC 1 values ranging from -9.01907 to -3.60154 and PC 2 values ranging from 0.27977 to 1.17514. In contrast, the "0.5 mM GABA" cluster occupies the upper left quadrant, with PC 1 values between 3.73708 and 8.8567, and PC 2 values from 0.40714 to 1.03281. Furthermore, the "1 mM GABA" cluster is found in the middle right quadrant, with PC 1 values ranging from -2.62036 to 2.6096 and PC 2 values between 1.09642 and 1.32916. On the other hand, the "0 mM ECT" cluster is situated in the lower right quadrant, encompassing data points with PC 1 values ranging from -9.25916 to -2.78645 and PC 2 values from -1.10333 to -0.50306. Moreover, the "0.25 mM ECT" cluster is observed in the middle left quadrant, with PC 1 values ranging from 2.79046 to -1.35643 and PC 2 values between -1.71727 and -0.72487. Lastly, the "0.50 mM ECT" cluster is positioned in the upper right quadrant, with PC 1 values ranging from 4.00189 to 9.30335 and PC 2 values from -1.01182 to -0.11442 (Fig. 11A).

The scores for each data point are given in two columns corresponding to PC 1 and PC 2. Additionally, the data points are labeled as "No AMF" and "AMF," indicating the presence or absence of Arbuscular Mycorrhizal Fungi (AMF) treatment. Looking at the plot, we can observe two separate clusters of data points representing the "No AMF" and "AMF" treatments. The "No AMF" cluster is situated in the lower left quadrant, with data points having negative values for PC 1 and PC 2. These points are spread across a range of PC 1 values from -9.25916 to -3.60154 and PC 2 values from -1.71727 to 1.17514. In contrast, the "AMF" cluster is located in the upper right quadrant, with data points having positive values for both PC 1 and PC 2. The PC 1 values for this cluster range from 0.1524 to 9.30335, while the PC 2 values vary from -1.31452 to 1.21826 (Fig. 11B).

The plot suggests several groups of variables that share similarities. For example, variables "Chlorophyll a (mg/g)" and "Total Chlorophyll (mg/g)" are closely related, as indicated by their small similarity value of 0.19291. Similarly, variables "Chlorophyll b (mg/g)" and "POD (U/g FW)" are linked with a similarity value of 0.51947. Another group of variables includes "Seed Dry Biomass (g/pot)" and "Root Dry Biomass (g/pot)," as well as "Stem Dry Biomass (g/pot)" and "Seed Cl (g/kg)." These pairs have similarity values of 0.92601 and 0.87348, respectively, suggesting a close relationship between them. Furthermore, variables related to elemental compositions such as "Leaf Na (g/kg)," "Leaf K (g/kg)," "Leaf Cl (g/kg)," "Leaf Ca (g/kg)," "Stem Na (g/kg)," "Stem K (g/kg)," "Stem Cl (g/kg)," "Stem Ca (g/kg)," "Seed Na (g/kg)," "Seed K (g/kg)," and "Seed Ca (g/kg)" form another cluster, indicating their similar patterns (Fig. 11C).

## Discussion

GABA, as a signaling molecule, promotes plant growth by stimulating cell division and elongation through the activation of GABA receptors and downstream signaling pathways [56, 57]. It enhances nutrient uptake, particularly for potassium (K), and improves nitrogen (N) assimilation [58]. Potassium is essential for the activation of various enzymes involved in plant growth, metabolism, and stress responses [59]. Under salinity stress, potassium enhances the activity of enzymes responsible for antioxidant defense, such as superoxide dismutase (SOD) and catalase (CAT) [60]. These enzymes scavenge reactive oxygen species (ROS) generated during salinity stress and protect plant cells from oxidative damage [60]. GABA also plays a vital role in osmotic stress protection by maintaining cellular osmotic balance [61]. It acts as an osmolyte, helping stabilize water potential in plant cells. On the other hand, potassium helps regulate stomatal opening and closure by influencing the movements of guard cells [62]. Adequate potassium levels maintain the turgor pressure in guard cells, allowing for efficient stomatal functioning and optimizing gas exchange, even under salinity stress conditions [63]. Furthermore, it also plays a vital role in maintaining ion homeostasis by competing with sodium for uptake and binding sites on cell membranes [64]. Adequate potassium supply helps reduce sodium uptake, preventing the toxic effects of sodium accumulation in plant cells [65]. Ectoine protects plants from osmotic stress by acting as a molecular stabilizer [66]. It safeguards proteins and cell membranes from damage caused by dehydration and high salinity [67]. Ectoine helps maintain ion homeostasis by balancing ion influx and efflux, ensuring optimal concentrations of essential ions like K and Ca [68]. Calcium plays a key role in maintaining cell membrane stability by binding to phospholipids and stabilizing the lipid bilayers [69]. This helps to prevent the leakage of ions and compounds from the cells, thereby preserving cell integrity. By stabilizing cell membranes, ectoine reduces membrane permeability and prevents electrolyte leakage [21]. Calcium ions act as crucial secondary messengers in signal transduction pathways in response to various stresses, including salinity stress [70, 71]. Changes in cytosolic calcium levels activate signaling cascades, leading to the expression of stress-responsive genes and the activation of stress defense mechanisms in plants [20, 72]. Moreover, ectoine enhances the activity of antioxidant enzymes, including SOD, POD, APX, and CAT, to scavenge ROS and counteract oxidative stress [22]. Antioxidants SOD and POD is an essential antioxidant enzyme that catalyzes the dismutation of superoxide radicals (O_2_-) into molecular oxygen (O_2_) and hydrogen peroxide (H_2_O_2_) [73]. This enzymatic reaction prevents the accumulation of superoxide radicals, which are highly reactive and can cause cellular damage [74, 75]. They also help to maintain cellular redox balance by converting superoxide radicals into less harmful molecules. APX is an important enzyme in the ascorbate–glutathione cycle, which is a key antioxidant system in plants [76–78]. APX reduces hydrogen peroxide (H_2_O_2_) using ascorbate (vitamin C) as the electron donor [74, 79–82]. By converting hydrogen peroxide into water, APX helps minimize oxidative stress and protects plant cells from damage caused by salinity stress [83]. In addition to the above, CAT is an enzyme that plays a crucial role in breaking down hydrogen peroxide (H_2_O_2_) into water and molecular oxygen (O_2_) [84]. It is a major antioxidant enzyme that helps detoxify hydrogen peroxide, which is a byproduct of various metabolic processes [85]. It contributes to maintaining cellular redox homeostasis and prevents the accumulation of hydrogen peroxide, which can be toxic to plant cells [86].

GABA's antioxidant properties allow it to scavenge harmful reactive oxygen species (ROS), reducing oxidative damage to chloroplasts where chlorophyll is located. Additionally, GABA can regulate stomatal closure, reducing water loss through transpiration and conserving water—a crucial factor in saline conditions [61]. Ectoine acts as a compatible solute, helping to stabilize cellular structures under stress conditions. This stability extends to chloroplasts, which contain chlorophyll. By protecting chloroplasts from damage, Ectoine indirectly supports chlorophyll synthesis and maintenance [67].

## Conclusion

In conclusion, γ-Aminobutyric acid (GABA) and ectoine (ECT) have the potential to alleviate the salinity stress in cotton when applied as foliar application. Treatment 0.5 mM GABA and 0.5 Mm ECT are the best application rates for the improvement in growth attributes of cotton cultivated under salinity stress. Both treatments i.e., 0.5 mM GABA and 0.5 Mm ECT have the potential to improve the cotton chlorophyll contents, gas exchange attributes, K and Ca concentration in leaf, stem, bur, and seeds. A significant decline in electrolyte leakage, Cl and Na of leaf, stem, bur, and seeds also validated the effectiveness of 0.5 mM GABA and 0.5 Mm ECT. Growers are recommended to use 0.5 mM GABA and 0.5 Mm ECT on cotton for the achievement of better growth and production under salinity stress. More investigations are suggested at the field level under different climatic conditions to declare 0.5 mM GABA and 0.5 Mm ECT as best treatments against salinity in cotton production.

## Study protocol must comply with relevant institutional, national, and international guidelines and legislation

Our experiment follows the with relevant institutional, national, and international guidelines and legislation.

## Data Availability

All data generated or analyzed during this study are included in this published article.
